# Editorial: Intracellular trafficking in lymphocytes: the role of inner crowds in determining cell fate

**DOI:** 10.3389/fimmu.2023.1212138

**Published:** 2023-05-17

**Authors:** Claudia M. Trujillo-Vargas

**Affiliations:** Grupo de Inmunodeficiencias Primarias, Facultad de Medicina, Universidad de Antioquia UdeA, Medellín, Colombia

**Keywords:** Familial hemophagocytic Lymphohistiocytosis, cytotoxic lymphocytes, natural killer (NK) cells, secretory lysosome trafficking, LRBA deficiency, autophagy, lysosomal-trafficking regulator (LYST), Chediak-Higashi Syndrome

Attending the meeting of the Latin American Society for Immunodeficiencies (LASID) in 2009, I started the journey on one of the most exciting scientific projects in my life. By that time, we were investigating patients with Familial Hemophagocytic Lymphohistiocytosis (FHL) due to perforin deficiency, in our laboratory in Medellín - Colombia (1). FHL is a life threatening immunodeficiency that, among other symptoms, causes fever, cytopenias and hepatosplenomegaly. Under normal circumstances, cytotoxic T lymphocytes and natural killer (NK) cells have the ability to “perforate” the cell membrane of virus-infected or tumor cell targets, propelling toxic substances into their cytoplasm, which ultimately induces their self-destruction by apoptosis. Due to genetic defects in *PRF1*, encoding perforin, these cells inefficiently clear target cells, which results in a cytokine storm with aberrant hyperactivation of a type of tissue phagocytic cells called histiocytes. We learnt that beyond the perforin deficiency, genetic defects in other molecules involved in the *secretory lysosome trafficking* confer a similar phenotype. In cytotoxic lymphocytes, secretory lysosomes are responsible for the transport of the toxic cargo to the cell membrane, where they fuse, delivering their content directly to their targets.

The process of inner communication between organelles such as secretory lysosomes in lymphocytes fascinated us and we spent several months trying to understand it. I travelled to LASID 2009 in Cartagena de Indias - Colombia, with the idea of networking to gain more insight into the mechanisms of organelle trafficking. In the lobby of the conference venue, I ran into Bodo Grimbacher, one of the world leaders in the characterization of monogenetic defects in immunity. After a short conversation but erring on the side of caution to express my specific scientific interest, I ended up asking him for a postdoctoral position in his lab. He promised to email me as soon as he had an available position, at that time at the Royal Free Hospital in London, UK. With this rather unspecific answer, I came back to Medellín. To my surprise, Prof. Grimbacher replied only some months later. His lab had some work in progress that might needed help for further characterization. Time went by fast and at the beginning of 2011 I was already in London, taking over the amazing work of Gabriela Lopez-Herrera on a gene termed *LRBA* (*LPS responsive beige-like anchor protein*). She was trying to characterize the consequences of the LRBA deficiency. Although LRBA is expressed in several immune cells including phagocytes and activated T and B cells, at that time, the pathophysiology of LRBA deficiency was unknown. Gabriela was screening for LRBA expression in patients with a compatible phenotype, evaluating the role of LRBA in B-cell apoptosis and searching for the molecular interacting partners of LRBA. But here comes the interesting part of the story for me, proving right what Rumi, a famous Sufi mystic said: “What you seek is seeking you”: Only by a huge coincidence, the most interesting aspect of this work was the suggested role of LRBA, based in previous research and comparative biology studies. LRBA is closely related to the *Lysosomal-trafficking regulator* (LYST) and was known to be an intracellular adaptor protein. LYST is a molecule that regulates the fusion of intracellular vesicles such as lysosomes. LYST deficiency causes Chediak-Higashi Syndrome, a disease that – as FHL – is related to defects in vesicular trafficking in lymphocytes. Submitting our work to *The American Journal of Human Genetics*, an anonymous reviewer suspected a defect in lysosome function and/or autophagy in our patients. Hence, we started evaluating autophagy in B-cells of LRBA-deficient patients and found it to be severely impaired. Lysosomes are essential in the degradation and recycling of intracellular material during autophagy. Defective autophagy leads to cell apoptosis. We finally published this impactful work, describing the first LRBA-deficient families and starting the characterization of the role of LRBA in B-cell apoptosis and autophagy (2).

Back in Medellín - Colombia, we soon obtained support to continue these collaborative studies. Overcoming all the limitations of doing research in a middle-income country, we were able to perform co-localization studies between LRBA and several endosomal and lysosomal markers in human mononuclear phagocytes (3). We found that LRBA was more strongly associated to early and late endosomes. Moreover, when cells were subjected to the autophagy flux, inhibitors of the phosphoinositide 3-kinase enzymatic pathway affected LRBA co-localization with LC3 (Microtubule-associated protein 1A/1B-light chain), an important marker of the autophagosomes. While we were immersed in this endeavor, Lo B et al. elucidated the important role of LRBA for the endosomal recycling of the molecule CTLA4 in T cells (4). However, after I left Prof. Grimbacher’s lab, another perseverant Colombian scientist took over the LRBA research, now at the Center for Chronic Immunodeficiency in Freiburg, Germany. Laura Gámez-Díaz had been a former student of us in Medellín. She and her team identified LRBA interaction partners at different stages of the autophagy process (5). They describe that LRBA interacts with the phosphoinositide 3-kinase regulatory subunit 4 (PIK3R4) and with the FYVE And Coiled-Coil Domain Autophagy Adaptor 1 (FYCO1).

That was the reason for my interest in the Research Topic: *Intracellular Trafficking in Lymphocytes: The Role of Inner Crowds in Determining Cell Fate*.

An outstanding review of diseases related to abnormalities in the vesicular budding and fusion of lymphocytes is included within this Research Topic (Vasanna and Dalal). We also include work about the polarization of centrosomes that, through a specialized array of stable detyrosinated microtubules, directs movement of organelles in the cytoplasm (Andrés-Delgado et al.). The role of the nuclear envelope is starting to be recognized. These membranes seem to be a receptacle of giant multi-protein complexes mediating antigen presentation, migration and fate in lymphocytes, among other functions in innate and adaptive immunity (Selezneva et al.). Finally, we presented outstanding work suggesting new mechanisms for the dynamic adaptation of intracellular trafficking in lymphocytes, based on alternative splicing (Ostwaldt et al.). Intensive research has to be performed to elucidate all the molecules and mechanisms that allow these “inner crowds” to move purposely and exchange cargo, guarantying “immune cell sustainibility”.

## Author contributions

The author confirms being the sole contributor of this work and has approved it for publication.
